# Sunitinib in Metastatic Renal Cell Carcinoma: A Systematic Review of UK Real World Data

**DOI:** 10.3389/fonc.2015.00195

**Published:** 2015-08-25

**Authors:** Miriam Argyropulo-Palmer, Aaron Jenkins, Davinder Singh Theti, James Larkin, David Montgomery

**Affiliations:** ^1^Pfizer Limited, Tadworth, UK; ^2^Department of Medical Oncology, The Royal Marsden Hospital, London, UK

**Keywords:** carcinoma, renal cell, clinical practice variations, drug reimbursement, real world data, sunitinib, United Kingdom

## Abstract

**Background:**

Real world data (RWD) are increasingly used to inform drug reimbursement decisions, but it is unclear how well outcomes from real world studies compare to those of clinical trials. This systematic review seeks to compare outcomes for sunitinib in routine UK clinical practice with the sunitinib registrational and expanded-access program clinical trials.

**Method:**

Systematic review of the real world published literature was undertaken. UK observational studies recording first- or second-line sunitinib efficacy were included. A qualitative summary of the results and comparison to the controlled clinical trials was conducted. Fifteen real world studies were included, 14 of which were only available as posters/presentations.

**Results:**

Real world study reporting quality was generally low, making comparisons with the clinical trials difficult. Practice relating to starting dose, dose modification, timing of therapy initiation, and other factors varied between centers. Median progression-free survival and adverse events were generally comparable to the clinical trial outcomes, but overall survival was not.

**Conclusion:**

There are few published data on sunitinib use in UK clinical practice. Studies are characterized by lack of peer reviewed publication and heterogeneity in design, reporting, and analysis. For use of RWD in the reimbursement setting, data collection and reporting will need to improve.

**Highlights:**

There are few published data on sunitinib use in UK clinical practice.

Studies are characterized by lack of peer reviewed publication and heterogeneity in design, reporting, and analysis.

Practice varies considerably between different UK centers.

Median progression-free survival and adverse events are generally comparable to the clinical trial outcomes, but overall survival is not.

For use of real world data in the reimbursement setting, data collection and reporting will need to improve.

## Introduction

Rising prevalence of, and improving survival from malignancy means that cost concerns form an increasingly important consideration in the allocation of healthcare resources. While value is increasingly discussed, there is no consensus as to how value should be measured as it pertains to new medicines. One key question is whether the randomized controlled clinical trial (RCT) provides the best basis for the assessment of the value that a medicine brings in clinical practice. In an attempt to address this, healthcare providers are increasingly looking to real world data (RWD) (1). While RCTs are the best source of evidence for determining the efficacy of a treatment in a specific population, there are perceived challenges in generalizing the results of these studies. RWD provides an opportunity to supplement evidence from RCTs with data relating to the use of the medicine in clinical practice. However, the greater susceptibility of RWD to sources of bias means that high quality studies are particularly important in providing an accurate assessment of how the drug works in clinical practice.

Sunitinib is a small molecule tyrosine kinase inhibitor (TKI) identified as a potent inhibitor of both vascular endothelial growth factor (VEGF) and platelet-derived growth factor (PDGF) receptors. Sunitinib demonstrated efficacy in the single arm phase II trials in cytokine refractory metastatic renal cell carcinoma (mRCC) patients (2, 3). From these results, sunitinib gained a conditional license for the treatment of mRCC, subsequently converted to a full license upon the results of the pivotal phase III trial (4).

In addition to the registrational studies, an expanded-access program (EAP) was initiated in 2005, at 246 sites in 52 countries across the world (5). The program was run to a tightly defined protocol, but in a wider range of centers and with less stringent entry criteria than the registrational trials. The primary objective was to provide sunitinib on a compassionate basis to patients with mRCC in countries where marketing authorization had not been granted, or who were ineligible for inclusion in the RCT. The authors also sought to provide additional understanding of the efficacy of sunitinib in patient sub groups less well represented in the registrational RCTs, particularly older patients, those with poorer performance status or brain metastases.

Clinical experience in mRCC in the UK has been substantial and there should therefore be a large evidence base upon which to establish the real world performance of sunitinib in the treatment of mRCC. This systematic review aims to identify the data pertaining to sunitinib use in UK clinical practice, focusing on safety and therapeutic effectiveness, and to compare these data to those in the registrational RCTs and the EAP. Quality of the reporting of these data will also be assessed.

## Materials and Methods

### Search strategy

All authors were involved in the development of the search strategy. We searched Medline, Medline (R) In-Process, EMBASE, EMBASE Daily Alerts, and BIOSIS Pre Reviews through OVID. Sunitinib was granted marketing authorization in 2006 and therefore studies published between 1 January 2006 and the 25 September 2013 were considered. We also searched relevant congresses; ASCO-Genitourinary, European Society of Medical Oncology, European International Kidney Cancer Symposium, and National Cancer Research Institute (NCRI), between January 2006 and September 2013. The complete search string used can be found in Table S1 in Supplementary Material. Citation screening was performed using Reference Manager 12 (Thomson Reuters). Finally, a number of real world studies were known to exist by the authors though not published in peer reviewed journals (6–9). These were included in the study and are available on request from corresponding author Davinder Singh Theti.

All identified citations had their titles and abstracts reviewed (if available) on first pass and those not considered relevant were excluded. Full papers were obtained for remaining references and assessed independently by two researchers. Disputes as to eligibility were discussed within the project team and resolved by consensus. All papers were reviewed according to the selection criteria given in Table 1. Reasons for excluding studies were documented.

**Table 1:** **Inclusion/exclusion criteria used in the systematic review of UK sunitinib real world studies**.

Criterion	Inclusion	Exclusion
Population	Adults ≥18 years with RCC classified as	Less than 18 years
	Advanced	Stage I or II
	Locally advanced	
		Stage III	
	Metastatic	
	Stage IV	
	Stage III/IV	
Intervention	First-line sunitinib	Studies of patients who were not receiving sunitinib as first line or post cytokine
	Treatment with sunitinib post cytokine	Sunitinib dose <25 mg or >75 mg/day
	Mixed population with ≤10% treated with non-first-line sunitinib or treated with prior anti-angiogenic therapy	Any regimen other than 4 weeks on 2 weeks off
	Sunitinib dose of 25–75 mg/day	Inclusion of other therapies for the treatment of RCC with sunitinib
	4 weeks on 2 weeks off regimen only	
Comparator	N/A	N/A
Outcomes	Effectiveness: PFS, OS, objective response rate (ORR), complete response (CR), partial response (PR), stable disease (SD), progressive disease (PD), time to progression (TTP), death rate (DR)	Studies that do not incorporate efficacy outcomes
	If efficacy data presented, then, patient reported outcomes (PROs): FACT-G, FKSI, EQ5D	
	Safety: incidence of all grade adverse events (AEs), incidence of grade 1–4 AEs	
Setting	UK Clinical practice	Non UK studies
Design	Non-RCT	Studies not pertaining to clinical practice
	Systematic review of non-RCT	RCT
	Observational studies	

### Data extraction

A pre-prepared data extraction table was created in Microsoft^®^ Excel. Outcomes extracted included median PFS (mPFS), median OS (mOS), PROs, and adverse events (AEs). We prospectively identified the following relevant prognostic or predictive factors for extraction along with other relevant information: prognostic score, age, sex, prior treatment, presence or absence of brain metastases, race, and smoking history. Surgical history was excluded as surgical resection is no longer considered a robust indication of performance status as it was in the pre TKI era. Data extraction was conducted independently by two researchers, with disputes resolved by consensus.

### Assessment of quality

Real world study quality was assessed independently by two different researchers. There is no consensus on a specific tool for the critical appraisal of real world studies. Real world studies often lack a control arm, and therefore, the authors selected the Chambers tool (10) as it does not penalize studies for this, thus reducing bias toward a low study quality. The number and pattern of questions answered with a “yes” indicates the quality of the study: “Good” = all eight questions answered yes; “satisfactory” = questions 2 and 4–7 answered with “yes”; “poor” if the answer is not “yes” to one or more of the questions required for a satisfactory rating (see Table S2 in Supplementary Material).

### Comparison of real world studies to the registrational sunitinib RCTs and EAP study

Assessment of the real world studies indicated that it was not appropriate for formal statistical analysis and consequently a qualitative approach to discussing the results in context of the results from the EAP and registrational studies was taken. Therefore, any comparisons between the two bodies of evidence are restricted to unadjusted, qualitative comparisons.

All real world studies were compared with the trial that had the most similar patient population. Studies with a mixed treatment population (including both first-line and second-line sunitinib) were compared against the EAP (5), which had no restrictive inclusion criteria concerning treatment line. Studies with treatment-naive patients were only compared against the phase III trial (4) and the treatment-naive subset in the EAP (5).

Two single arm phase II studies were conducted with sunitinib. The first study enrolled a total of 63 mRCC patients who had failed prior cytokine treatment (3). The second study enrolled a total of 106 clear cell mRCC patients who had failed prior cytokine treatment (2). Both reported similar outcomes. The larger 2007 study only was used for comparison in this systematic review. Studies that analyzed prior cytokine patients separately were compared against this phase II trial and the prior cytokine subset in the EAP. The EAP is used as a comparator for all real world studies, as analysis of treatment-naive and prior cytokine patients is reported both separately and together.

## Results

**Figure 1:**
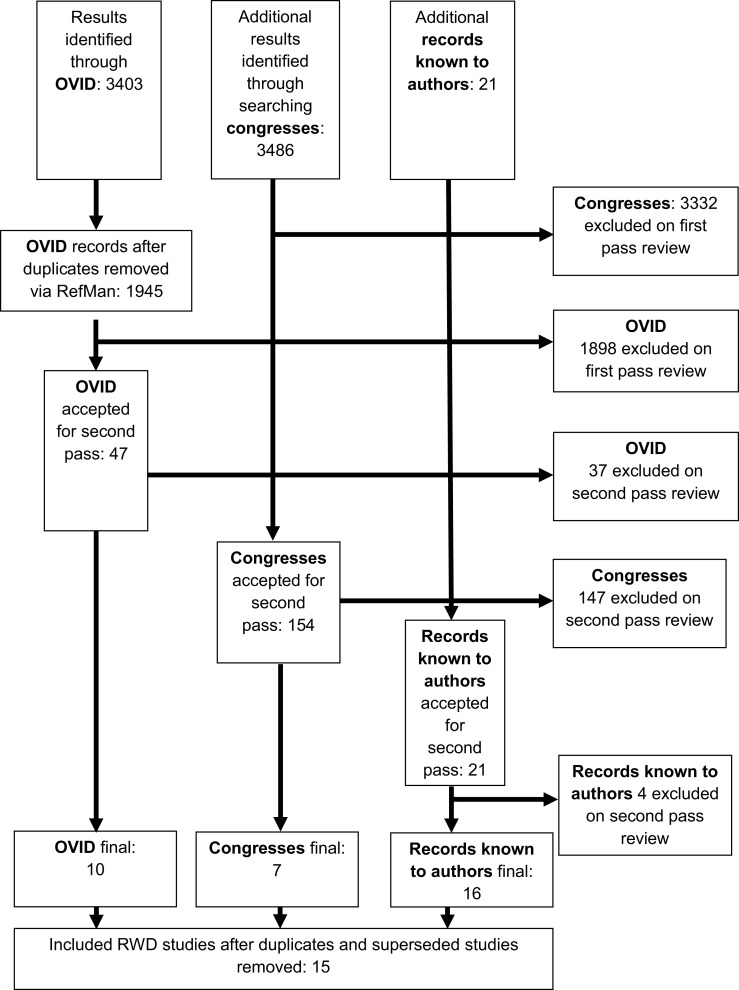
**CONSORT study flow diagram for the identification and selection of studies for this systematic review**.

In total, 6,910 references were identified. Three thousand four hundred three through the OVID database search, 3,486 through the search of congresses. Twenty-one records of real world sunitinib data were known to the authors prior to searching. A summary is provided in the CONSORT flow diagram (Figure 1). Of the 3,403 references obtained through OVID, 47 references remained after duplicates were removed and first pass review was conducted. Thirty-seven were excluded after further examination of the full text. Of these, 21 were excluded as they were not UK studies, 1 was excluded due to inclusion of other adjuvant therapies for the treatment of RCC with sunitinib, 7 were case reports, and 8 lacked efficacy outcomes.

Of the total 3,486 references obtained from congresses, 154 references remained after first pass review. One hundred forty-seven references were excluded after further examination of the full text. Of these, 141 were excluded as they were not UK studies, 1 for reporting RCT data, 3 for lacking outcomes of relevance, and 2 for reporting on a different tumor type.

All 21 previously known references were retained for second pass review, at which point 4 references were subsequently excluded each for a different reason; data from outside of UK clinical practice, lack of efficacy data, case report, and lack of sunitinib data. After second pass review, 16 studies remained in total. Several hospitals reported data more than once in different formats. All references that recorded duplicate data were excluded; 15 studies remained for inclusion in the analysis.

### Study characteristics

A summary of the key study characteristics of the registrational and EAP studies are given in Table 2 (2, 3, 5). Of note is how between them, the trials represent cytokine refractory (phase II) and treatment-naive (phase III) patients or both (EAP). Between them, the clinical trials provide diverse population subsets to compare with the real world studies.

**Table 2:** **Summary of the sunitinib registrational and EAP studies used as comparators in this systematic review**.

Study	Patient no.	Study design	Reported outcomes
Motzer et al. (2), Phase II	106	Single arm trial of sunitinib in second-line cytokine refractory patients	Endpoints: primary – ORR, secondary – TTP, PFS, OS, duration of response
Motzer et al. (4), phase III	750	Randomized, controlled study of sunitinib vs. interferon alpha in treatment-naive patients	Endpoints: primary – PFS, secondary – ORR, OS, safety
Gore et al. (5), EAP	4564	Observational study of sunitinib in trial ineligible mRCC patients in countries without licensed access. Population included first and second line patients	PFS, OS, ORR in the subgroups: over 65, ECOG PS ≥2, brain metastases, prior cytokine treatment, non-clear cell histology. Safety evaluated in all patients

Baseline characteristics of the real world studies are given in Table 3. The number of patients per study ranged from 14 to 395. Ten studies included analysis of first line, treatment-naive patients (8, 11–15). Two studies included a separate analysis of all patients and second line, prior cytokine patients (14, 15). One study analyzed second-line (prior cytokine) patients only (6). Six studies included analysis of a mixture of first- and second-line treatment (14–19).

**Table 3:** **Baseline characteristics of patients in the sunitinib real world studies and registrational/EAP studies used as comparators in this systematic review**.

Study	Patient no.	Prognostic score or PS	Brain metastases (%)	Age as given (range if given)	Sex (% male)
Motzer et al. (2), phase II	106	MSKCC: F = 57.5%, I = 38.7%, P = 3.8%	0.0	Median 56 years (32–79)	M 63
Motzer et al. (4), phase III	750	MSKCC: F = 38%, I = 56%, P = 6%, PS: 0 = 62%, 1 = 38%	0.0	Median 62 years (27–87)	M 71
Gore et al. (5), EAP	4564	MSKCC: F = 36%, I = 44%, P = 9%, NR = 11%, PS: 0 = 42%, 1 = 43%, 2 = 12%, 3 = 2%, 4 <1%	7.0	Median 59 years (19–89)	M 74
Coward et al. (20)	62	NR	NR	≤70, 61%; ≥70, 39%	NR
Fisher et al. (15)	62 TN = 39, PT = 18	MSKCC: F = 62.5%, I = 35.9%, P = 1.6%	NR	Median 57 years	M 75
Galvis et al. (14)	395 TN = 262 PT = 133	PS: 0–1 = 81%, >1 = 19%	NR	≤60 years = 30%; 61–70 years = 42%; ≥70 years = 28%	M 66
Goranova et al. (17)	129	PS: 0 = 33%, 1 = 51%, 2 = 16%	NR	Median 63 years (21–87)	M 67
Liberatoscioli et al. (6)	31	MSKCC: F = 61.3%, I = 25.8%, P = 12.3%; PS: 0 = 32.3%, 1 = 38.7%, 2 = 25.8%, 3 = 3.2%	6.5%	Median 58 years (38–71)	M 74.3
Maclennan et al. (12)	87	PS: 0 = 22%, 1 = 59%, 2 = 18%	16.0%	Median 58 years (39–78)	M 72
MacLeod et al. (11)	73	PS: 0 = 18%, 1 = 18%, 2 = 5%, NR = 59%	NR	Median 62	M 74
Miscoria et al. (16)	141	Heng: F = 19.9%, I = 53.2%, P = 26.9%; PS: 0–1 = 78.7%, 2–3 = 21.3%	NR	Median 61 (33–86)	M 75
Mullard et al. (21)	42	Heng: F = 43%, I = 38%, P = 19% PS: 0–1 = 91%	NR	Median 61 years (43% >65 years)	NR
Sparrow (9)	14	PS: 0 = 28%, 1 = 72%	0.0	Median 65 years (53–80)	M 64
Susnerwala (8)	35	PS: 0 = 3%, 1 = 80%, 2 = 14%, NR = 3%	0.0	NR	M 60

*NR, not recorded; TN, treatment naïve; PT, pre-treated. MSKCC, Memorial Sloane Kettering Cancer Centre. F, favorable; I, intermediate; P, poor. Studies by James et al. (18), Sim and Hayward (7), Visvardis et al. (13), and Wagstaff et al. (19) were omitted from this table as they could not be compared directly to the registrational studies or EAP*.

Thirteen studies reported PFS or OS data. Despite reporting efficacy data, three of these studies were not comparable against any of the trials, as they investigated subgroups not represented in the registrational or EAP studies, e.g., receiving treatment vs. not receiving treatment (18), outcomes by plasma thyroid stimulating hormone levels (7), and Furhman grading (13). Wagstaff et al. (19) was not compared as the study was restricted to AEs and ORR.

### Quality assessment

Methodological quality was rated as poor according to the Chambers tool for all real world studies. Most studies were recorded in poster or abstract form and so lacked sufficient detail to score yes to all questions. The full quality assessment for each study is given in Table S2 in Supplementary Material.

### Comparison of mPFS and mOS of real world studies with the registrational and EAP studies

Results for mPFS and mOS from the real world studies are summarized in Table 4, along with unadjusted comparisons against the clinical trial of most relevance. The phase II study (2) reported mPFS of 8.8 months (95% CI 7.8–13.5) and mOS of 23.9 months (95% CI 14.1–30.7). The phase III registration study (4) reported mPFS of 11 months with sunitinib (95% CI 11.0–13.0) compared with mPFS of 5 months with interferon (IFN) alpha (95% CI 4.0–6.0). Median OS was 26.4 months in the sunitinib arm (95% CI 23.0–32.9) compared with 21.8 months in the IFN-alpha arm (95% CI 17.9–26.9). The EAP (5) reported a similar mPFS of 10.9 months (95% CI 10.3–11.2) for all patients (10.8 months in patients with prior cytokine treatment and 11.1 months in treatment-naive patients). Median OS was lower in the EAP than in the phase III registration study, at 18.4 months (95% CI 17.4–19.2) for all patients (18.4 months in patients with prior cytokine treatment and 18.1 months in treatment-naive patients).

**Table 4:** **Comparison of real world studies compared to relevant registrational and EAP studies**.

Study (*N* = population)	Median PFS in months		Median OS in months	
	Real world study	Comparator study	Real world study	Comparator study
Coward et al. (20) (*N* = 62), treatment-naive population	NR	NA	23	Phase III 26.4 EAP-TN 18.1
Fisher et al. (15) (*N* = 18), pre-treated population	6.7 (95% CI 0.7–12.7)	Phase II 8.8 EAP-PT 10.8	37.6 (95% CI 2.6–72.5)	Phase II 23.9 EAP-PT 18.4
Fisher et al. (15) (*N* = 39), treatment-naive population	9 (95% CI 8.1–9.9)	Phase III 11.0 EAP-TN 11.1	17.4 (95% CI 11.6–23.2)	Phase III 26.4 EAP-TN 18.1
Galvis et al. (14) (*N* = 395), mixed population	11.0	EAP-M 10.9	18.0	EAP-M 18.4
Galvis et al. (14) (*N* = 133), pre-treated population	NR	NA	20.5	Phase II 23.9 EAP-PT 18.4
Galvis et al. (14) (*N* = 262), treatment-naive population	NR	NA	18.6	Phase III 26.4 EAP-TN 18.1
Goranova et al. (17) (*N* = 129), mixed population	10	EAP-M 10.9	17.0	EAP-M 18.4
Liberatoscioli et al. (6) (*N* = 31), pre-treatment population	10.9	Phase II 8.8 EAP-PT 10.8	26.3	Phase II 23.9 EAP-PT 18.4
Maclennan et al. (12) (*N* = 87), treatment-naive population	8.9	Phase III 11 EAP-TN 11.1	14.7	Phase II 23.9 EAP-PT 18.4
MacLeod et al. (11) (*N* = 73), treatment-naive population	NR	NA	14.4	Phase II 26.4 EAP-TN 18.1
Miscoria et al. (16) (*N* = 141), mixed population	10.8	EAP-M 10.9	18.4	EAP-M 18.4
Mullard et al. (21) (*N* = 42), treatment-naive population	NR	NA	10.0	Phase III 26.4 EAP-TN 18.1
Sparrow (9) (*N* = 14), mixed population	9.0 (95% CI 8.1–10.0)	EAP-M 10.9	25.2 (95% CI 8.0–42.2)	EAP-M 18.4
Sparrow (9) (*N* = 9), treatment-naive population	NR	NA	10.5 (range 3–32)	Phase III 26.4 EAP-TN 18.1
Susnerwala (8) (*N* = 34), treatment-naive population	9.0	Phase III 11.0 EAP-TN 11.1	20.0	Phase III 26.4 EAP-TN 18.1

*EAP-M, EAP mixed population; EAP-T N, EAP treatment naïve population. EAP-PT, EAP pre-treated population; NR, not recorded; NA, not applicable. Studies by James et al. (18); Sim and Hayward (7), Visvardis et al. (13), and Wagstaff et al. (19) were omitted from this table as they could not be compared directly to the registrational studies or EAP*.

Five real world studies analyzed a mixed patient population [first-line and prior cytokine; (14, 16–19)]. Of these, only three studies reported mPFS and mOS. In each of these studies, mPFS was similar to that reported in the EAP for all patients, at 10 months (17), 11 months (14), and 10.8 months (16). Median OS was also similar, at 17 months (17), 18 months (14), and 18.4 months (16).

Of the eight real world studies that included treatment-naive patients only, all included some PFS or OS analysis (7–9, 11–13, 20, 21), but only two reported mPFS suitable for comparison. The mPFS in these two studies was similar to that reported in the phase III study and EAP, at 8.9 months (12) and 9 months (8). Seven studies reported mOS suitable for comparison, which varied widely, ranging from 10 months (21) to 23 months (20).

Of the three studies that analyzed therapeutic effectiveness in cytokine refractory patients (6, 14, 15), two reported on mPFS (6, 15). Both reported a similar mPFS compared to the comparative trials (phase II and EAP) at 9 months (15) and 10.9 months (6). All three studies reported on mOS, two of which were lower in the real world setting compared to the phase II study, at 17.4 months (15) and 20.5 months (14).

### Safety

Relative to the registrational studies and EAP, no new AEs were reported in any of the real world studies.

### Outcomes related to sunitinib access

One study (18) investigated the effect of access to first-line treatment (sunitinib or sorafenib) on OS. Prior to approval by NICE, access for sunitinib and sorafenib was determined by local funding decisions and resulted in variable access across England and Wales. James et al.’s data showed that patients who received treatment with sunitinib or sorafenib compared to those who did not had a longer OS (hazard ratio = 0.46, 95% CI 0.21–1.01, *p* = 0.05). The OS advantage was similar between patients receiving sunitinib (hazard ratio = 0.49, 95% CI 0.18–1.36, *p* = 0.17) or sorafenib (hazard ratio = 0.44, 95% CI 0.11–1.69, *p* = 0.21).

### Comparison of treatment center on outcomes

Goranova et al. (17) was the only study to compare mPFS and mOS between two cancer centers [Northern Centre for Cancer Care (NCCC) and James Cook University Hospital (JCUH)]. Along with outcome data, the study compared variables at these two centers including dose reduction at outset, WHO PS, and dose delay of 4 weeks or more. mPFS was longer among patients treated at NCCC compared with JCUH (12 and 6 months, respectively). Median OS was also higher at NCCC compared with JCUH, with NCCC documenting mOS of 23 months compared to 14 months at JCUH. The distribution of patients by PS was similar between the two centers, and no other comparative information on baseline characteristics was provided. Both initiation of treatment at reduced dose and use of treatment breaks differed between the centers. NCCC started fewer patients on a reduced dose (six patients) compared with JCUH (22 patients). Similarly, NCCC delayed dose in fewer patients than JCUH (8 and 19 patients, respectively). Patients who had undergone treatment delay experienced significantly better mPFS and numerically better mOS than those who did not (mPFS: 17 vs. 6 months, respectively, *p* = 0.0265 and mOS: 23 vs. 14 months, respectively, *p* = 0.0705).

## Discussion

There are very few published reports of sunitinib real world use in the UK. Of the studies, which have been conducted, only two were published in full text peer reviewed journals at the time of performing this study, the majority appearing in abstract form. The majority of studies achieved a score of “poor” on the Chambers quality assessment tool, mainly through lack of reporting of basic methodological detail. None of the studies reported loss to follow up or described the methodology used for defining progression or response and it is unclear whether RECIST criteria was appropriately applied and whether response was investigator assessed by blinded radiological review or determined in some other way. Several of the studies were designed to answer quite specific questions and had selected patient groups. There was extensive heterogeneity in the patients treated and in the clinical practices described, making it difficult to extrapolate the results of these studies to a wider patient group.

### Therapeutic effectiveness in the real world

The data, which have been published, and notwithstanding the caveats described above regarding interpretation of those data, suggest that sunitinib is performing comparably in the real world setting to the registrational and EAP studies with regards to PFS. mPFS of treatment-naive patients in the real world was consistently similar to that reported in the phase III trial (despite including patients of a poorer prognosis) and EAP. The impact on OS was more interesting. Of the treatment-naive population, the lowest mOS reported was 10 months (21), lower than the mPFS recorded in the phase III trial or EAP. In contrast, Coward et al. (20) reported mOS of 23 months, similar to that seen in the phase III study, and greater than reported in the EAP. Furthermore, given the strict inclusion criteria in the phase III trial (ECOG PS 0–1 only) compared with the real world studies (the majority included a considerable proportion of patients of PS ≥2; see Table 4) and the association between poor PS and poorer outcome, these results demonstrate that the efficacy reported in the pivotal trial can be reproduced in clinical practice.

Why the OS was not more consistently reproduced in the real world when PFS is, is not clear. Assuming that the data are accurate, it implies a shorter time from progression to death in some centers than in others or the registrational studies and EAP. There are a number of possibilities, which include definition of progression being different from that used in clinical trials (and so reported PFS being artificially longer in the RWD studies), patients being sicker at progression, or perhaps poorer access to second-line treatment options (such as funded second-line treatments or clinical trials) than in the registrational studies.

In the phase III study, 56% of the sunitinib patients received post-study treatment (4). Only two of the real world studies reported proportion of patients that received second-line treatment and both studies reported much lower proportions than in the phase III study (Susnerwala – 20% and Mullard et al. – 12%). In the UK, between 2007 and 2008, only 2.3% of renal cancer patients were entered into clinical trials of all types; both localized and metastatic (22) annual report and most of the RWD recorded in the current study pertains to patients treated at a time when no funded second-line agents were available in the UK. This is therefore a plausible reason for the consistently lower mOS in the real world studies compared to the phase III study. Unfortunately, most of the studies lacked sufficient reporting of baseline patient characteristics and of subsequent treatments given to allow us to reach a conclusion.

### Treatment heterogeneity between centers

Dose intensity is an important determinant of therapeutic effect. Goranova et al. (17) reported that patients who were started on a lower dose of sunitinib had lower mPFS than those who were started at 50 mg, the recommended starting dose. That study reported patients from two centers where, despite broadly similar patient demographics and close geographical proximity, starting at lower dose was more prevalent in one center that also reported poorer outcomes. Higher plasma exposure to sunitinib has been shown to be associated with longer TTP and mOS compared with lower exposure (23). This implies that some of these patients who were started at a lower dose were not receiving optimal exposure. The authors also question whether they could achieve better results, and highlight initiation dose and willingness to dose escalate to find the optimal treatment dose as two areas that could be further optimized; implicitly to lessen the variation in outcome for patients between these geographically close centers.

### Use of real world data to inform drug reimbursement

Real world data are increasingly being called upon to guide clinical practice and to help payers refine their assessment of “value.” The growing importance of RWD has led to the establishment of novel initiatives, such as the UK’s systemic anti-cancer therapy (SACT) dataset and linkage to other databases, such as the National Cancer Registration Service (NCRS) database. This, in part, is the consequence of a recognized unmet need for quality RWD that can be accessed as an aid to making well-informed funding decisions and improving service provision where necessary. NHS England’s “commissioning through evaluation” program provides a framework to make available specialized treatments, which are not routinely available due to inadequate evidence of clinical or cost effectiveness. Data will be collected prospectively with a view to informing the decision regarding routine commissioning (24). The national Cancer Drugs Fund in England may also adopt this approach. The UK Department of Health has suggested that the collected RWD will be used to negotiate pricing discounts with pharmaceutical companies (25). These uses of RWD may prove controversial. Reimbursement decisions in particular require robust, high quality comparative data, which provide some indication as to the relative effect of new vs. established treatments. An RCT provides such comparative evidence, but a single arm real world study, or even prospective collection of data on the effectiveness of a new medicine in the real world, does not.

End points used in clinical trials may be impractical in the real world setting. In oncology in particular, PFS is commonly used as the primary end point for registrational purposes, but PFS will not be collected under the NCRS. Less rigorous scanning requirements in the real world may make any comparisons less meaningful. It is likely that these reimbursement decisions will rely on OS as an end point.

As we can see from this review, in small data sets, PFS was fairly reproducible but OS was not. Had this systematic review assessed OS alone, it would have been difficult to conclude anything other than that sunitinib was not performing as well in the real world, but real world PFS data in this review refute that. If anything, it would appear that a combination of a lack of subsequent treatment options and perhaps slightly poorer patient prognostic characteristics are to blame for the lower survival, although this latter point is difficult to state definitively as patient characteristics were often not adequately described in the included analyses.

## Summary

Real world data will increasingly be used to guide reimbursement decisions, but the interpretation of those data is difficult due to often inadequately described methodology and substantial heterogeneity in clinical practice. While PFS in the treatment-naive populations from real world studies was consistent with the phase III trial, OS varied considerably. The variability in OS could be due to a number of factors, including access to second-line treatment or other differences in clinical practice or patient demographic features. Unfortunately, the reported real world studies were insufficiently detailed to allow further exploration of the reasons underpinning this heterogeneity, but do raise significant concerns that health technology assessments underpinned by naive comparisons of RWD OS vs. survival in clinical trials could be heavily biased. These problems often exist with published observational data and the impact that poor reporting has on usability is well known. An agreed set of reporting criteria have been produced [the STROBE statement; von Elm et al. (26)] to provide further guidance on optimizing reporting of observational data. This review highlights the need for authors to adhere to this, especially when presenting findings in posters or abstracts. Whole system wide data registries, such as those proposed in the UK, could be a powerful tool and may go some way to ensuring comparable populations and reducing heterogeneity. That said, some heterogeneity of patient features and clinical practice will be inevitable and it is critical, for these databases to be of use, that comprehensive data are captured on individuals, incorporating outcomes from the whole treatment path from diagnosis to cure or death. Only in this way will investigators seeking to make assessments of the relative effectiveness of medicines have the tools to do so.

## Conflict of Interest Statement

This systematic review was funded by Pfizer Ltd. Aaron Jenkins, Davinder Singh Theti, and David Montgomery are paid employees of Pfizer Ltd. and shareholders of Pfizer Inc. At the time of performing this study, Miriam Argyropulo-Palmer was a paid industrial placement student at Pfizer Ltd. and David Montgomery was a member of the National Cancer Drugs Fund review panel. James Larkin has no conflicts of interest relevant to this study.

## Supplementary Material

The Supplementary Material for this article can be found online at http://journal.frontiersin.org/article/10.3389/fonc.2015.00195

## References

[1] Association of the British Pharmaceutical Industry (ABPI). Demonstrating Value with Real World Data. (2011). Available from: http://www.abpi.org.uk/our-work/library/guidelines/Documents/2011-06-13%20ABPI%20guidance%20-%20Demonstrating%20value%20with%20real%20world%20data.pdf

[2] Motzer RJ Michaelson MD Rosenberg J Bukowski RM Curti BD George DJ Sunitinib efficacy against advanced renal cell carcinoma. J Urol (2007) 178(5):1883–7. 10.1016/j.juro.2007.07.030 17868732

[3] Motzer RJ Michaelson MD Redman BG Hudes GR Wilding G Figlin RA Activity of SU11248, a multitargeted inhibitor of vascular endothelial growth factor receptor and platelet-derived growth factor receptor, in patients with metastatic renal cell carcinoma. J Clin Oncol (2006) 24(1):16–24. 10.1200/JCO.2005.02.2574 16330672

[4] Motzer RJ Hutson TE Tomczak P Michaelson MD Bukowski RM Oudard S Overall survival and updated results for sunitinib compared with interferon alfa in patients with metastatic renal cell carcinoma. J Clin Oncol (2009) 27(22):3584–90. 10.1200/JCO.2008.20.1293 19487381 PMC3646307

[5] Gore ME Szczylik C Porta C Bracarda S Bjarnason GA Oudard S Safety and efficacy of sunitinib for metastatic renal-cell carcinoma: an expanded-access trial. Lancet Oncol (2009) 10(8):757–63. 10.1016/S1470-2045(09)70162-7 19615940

[6] Liberatoscioli C Galvis V Spencer-Shaw A Hawkins RE Treatment Outcomes of Second Line Tyrosine Kinase Inhibitors After High Dose IL-2 in Patients With Metastatic Clear Cell Renal Cell Carcinoma. (2011). [Poster session; available on request from corresponding author Davinder Singh Theti].

[7] Sim S Hayward K Managing Hypothyroidism in Metastatic Renal Cell Carcinoma Patients Receiving Sunitinib. (2011). [Poster session; available on request from corresponding author Davinder Singh Theti].

[8] Susnerwala S Sutent in Metastatic Renal Cell Carcinoma at Blackpool Victoria Hospital. (2011). [Poster session; available on request from corresponding author Davinder Singh Theti].

[9] Sparrow. Audit of Patients With Advanced Renal Cancer Receiving Sunitinib. (2011). [Poster session; available on request from corresponding author Davinder Singh Theti].

[10] Chambers D Rodgers M Woolacott N Methods of systematic reviews and meta-analysis. J Clin Epidemiol (2009) 62:1253–60. 10.1016/j.jclinepi.2008.12.010 19349144

[11] MacLeod N Laskey J Pace L Aitchison M Jones R Medicines use review in renal cancer: how are we doing? What could we do better? J Clin Oncol (2012) 30(5 Suppl):458.

[12] Maclennan M Connolly K Malik J Mitchell B O’Dea R Law A Hypothyroidism and Hypertension as Markers of Outcome in the Treatment With Sunitinib Malate for Metastatic Renal Cell Carcinoma: The Edinburgh Experience. NCRI Annual Meeting. (2012). Available from: http://conference.ncri.org.uk/abstracts/2012/abstracts/A86.html

[13] Visvardis E Waxman J Savage P Differential response of patients with metastatic renal cell carcinoma to sunitinib according to the Fuhrman grading of their targeted tumor. J Clin Oncol (2011) 29(15 Suppl):e15056.

[14] Galvis V Lawrence D Howell M Thistlethwaite F Hawkins RE Outcomes of patients with metastatic renal cell cancer treated with sunitinib in clinical practice at a reference cancer centre in Manchester, United Kingdom. J Clin Oncol (2013) 31(6 Suppl):477.

[15] Fisher RA Pender A Thillai K Chowdhury S Pickering LM St.Rose S Observation prior to systemic therapy in patients with metastatic renal cell carcinoma in the kinase inhibitor era. J Clin Oncol (2011) 29(15 Suppl):4630.

[16] Miscoria M Tew A Baijal S Pirrie S Holmes S James N Dose management and toxicities in 137 unselected patients with advanced kidney cancer treated with sunitinib. Ann Oncol (2010) 21(8 Suppl):vii298.

[17] Goranova RA Goranov BB Pedley ID Azzabi A Humphreys A Mcmenemin R U.K. experience of sunitinib malate in the treatment of metastatic renal cell carcinoma: predictors of response from clinical practice. J Clin Oncol (2012) 30(5 Suppl):455.

[18] James N Pascoe J Zachariah A Ray D Oldroyd A Parry H Effect of the UK postcode lottery on survival of patients with metastatic renal cancer: an audit of outcomes in patients with metastatic renal cancer suitable for treatment with tyrosine kinase inhibitors. Clin Oncol (R Coll Radiol) (2009) 21(8):610–6. 10.1016/j.clon.2009.06.007 19695849

[19] Wagstaff J Hawkins RE Nathan PD Sarda SP Vekeman F Korves C Sunitinib (SU) treatment (trx) patterns and toxicity in patients (pts) with advanced renal cell carcinoma (RCC) in United Kingdom (UK). J Clin Oncol (2011) 29(15 Suppl):e15150.

[20] Coward JIG Larbi ED Pandha HS Michael A. The effect of age on first-line sunitinib treatment in patients with renal cell carcinoma (RCC). J Clin Oncol (2011) 29(15 Suppl):e15096.26011553

[21] Mullard A Purcell S Carser J Griffiths R Sunitinib Therapy for Metastatic Renal Cell Carcinoma – the Mersey Experience, NCRI Annual Meeting. (2012). Available from: http://conference.ncri.org.uk/abstracts/2012/abstracts/A10.html

[22] National Cancer Research Institute (NCRI). Renal Cancer Clinical Studies Group Annual Report. (2013). Available from: http://csg.ncri.org.uk/wp-content/uploads/2013/10/NCRI-CSG_Renal_Annual-Report_2012-2013.pdf

[23] Houk B Bello CL Poland B Rosan LS Demetri GD Motzer RJ. Relationship between exposure to sunitinib and efficacy and tolerability endpoints in patients with cancer: results of a pharmacokinetic/pharmacodynamic meta-analysis. Cancer Chemother Pharmacol (2010) 66:357–71. 10.1007/s00280-009-1170-y 19967539

[24] NHS England. NHS England Invites Specialised Services Providers to Take Part in its Innovative New Programme ‘Commissioning Through Evaluation’. (2013). Available from: http://www.england.nhs.uk/2013/09/26/com-through-eval

[25] Department of Health. Thousands More Patients to Benefit From Additional £160 Million for Cancer Drugs. (2014). Available from: https://www.gov.uk/government/news/thousands-more-patients-to-benefit-from-additional-160-million-for-cancer-drugs

[26] von Elm E Egger M Altman DG Pocock SJ Gotasche PC vandenbroucke JP Strengthening the reporting of observational studies in epidemiology (STROBE) statement: guidelines for reporting observational studies. Br Med J (2007) 335:806–8. 10.1136/bmj.39335.541782.AD 17947786 PMC2034723

