# Supplementary material for: Sunitinib in Metastatic Renal Cell Carcinoma: A Systematic Review of UK Real World Data
Source: Front Oncol. 2015 Aug 25;5:195. doi: 10.3389/fonc.2015.00195 (PMC4548684; doi:10.3389/fonc.2015.00195)
Supplement: Supplementary file 1 [file Table_1.PDF]

#	Search Statement	Results
1	exp Carcinoma, Renal Cell/	66276
2	Kidney Neoplasms/	85748
3	exp *kidney cancer/	90862
4	carcinoma, renal cell.ti,ab.	192
5	((kidney$ or renal or nephro$) adj2 (cancer$ or carcinom$ or adenocarcinom$ or neoplasm$ or tumo?r$ or sarcoma$ or adenom$)).ti,ab.	135377
6	mrcc.ti,ab.	2691
7	renal cell.ti,ab.	84791
8	arcc.ti,ab.	270
9	rcc.ti,ab.	27065
10	Nephroblastom$.ti,ab.	5553
11	Papillary.ti,ab.	119292
12	Oncocytoma.ti,ab.	4792
13	((oxyphil$ or Hu?rthle) adj2 (tumo?r$ or adenoma$)).ti,ab.	1681
14	(Collecting duct$ or collecting tubule$).ti,ab.	28796
15	Transitional.ti,ab.	77905
16	Sarcomatoid.ti,ab.	5977
17	(Chromophobe or chromofobe).ti,ab.	5049
18	Medullary.ti,ab.	104545
19	Hypernephroid.ti,ab.	513
20	Hypernephroma$.ti,ab.	3103
21	1 or 2 or 3 or 4 or 5 or 6 or 7 or 8 or 9 or 10 or 11 or 12 or 13 or 14 or 15 or 16 or 17 or 18 or 19 or 20	509973
22	SU11248.ti,ab.	403
23	*sunitinib/	2680
24	Sunitinib.ti,ab.	9771
25	SU-11248.ti,ab.	67
26	SU011248.ti,ab.	25
27	"SU 11248".ti,ab.	67
28	SU-011248.ti,ab.	7
29	"SU 011248".ti,ab.	7
30	sutent.ti,ab.	425
31	22 or 23 or 24 or 25 or 26 or 27 or 28 or 29 or 30	10401
32	21 and 31	5296
33	limit 32 to English	4896
34	limit 33 to human [Limit not valid in Embase Weekly Alerts; records were retained]	4221
35	limit 34 to humans [Limit not valid in Embase Weekly Alerts; records were retained]	4221
36	limit 35 to yr="2006 -Current"	4168
37	randomized controlled clinical trial.kw.	1568
38	Controlled clinical trial.kw.	5567
39	Randomi?ed.ab.	1021105

40	Placebo.ab.	461587
41	Clinical trials as topic.sh.	174545
42	Randomly.ab.	618583
43	Trial.ti.	390959
44	37 or 38 or 39 or 40 or 41 or 42 or 43	2072426
45	36 and 44	765
46	36 not 45	3403
47	remove duplicates from 46	1945

Supplementary Table 1. Search string for identification of UK real world studies for inclusion in this systematic review.

Databases searched - Ovid MEDLINE(R), BImOSIS Previews, Embase Weekly Alerts, Embase.
