# Supplementary material for: Sunitinib in Metastatic Renal Cell Carcinoma: A Systematic Review of UK Real World Data
Source: Front Oncol. 2015 Aug 25;5:195. doi: 10.3389/fonc.2015.00195 (PMC4548684; doi:10.3389/fonc.2015.00195)
Supplement: Supplementary file 2 [file Table_2.PDF]

	Chambers Assessment							
Real World Study	1. Were selection/ eligibility criteria adequately reported?	2. Was the selected population representative of that seen in normal practice?	3. Was an appropriate measure of variability reported?	4. Was loss to follow-up reported or explained?	5. Were at least 90% of those included at baseline followed up?	6. Were patients recruited prospectively?	7. Were patients recruited consecutively?	8. Did the study report relevant prognostic factors?
Coward *et al.,* 2011	No	Yes	No	No	No	No	Cannot determine	No
Fisher *et al.,* 2011	Yes	No	Yes	No	No statement	No	Cannot determine	Yes
Galvis *et al.,* 2013	Yes	Yes	No	Yes	Yes	No	Cannot determine	Yes
Goranova *et al.,* 2012	No	Yes	No	No	No statement	No	Cannot determine	yes
James *et al.,* 2009	No	Yes	Yes	No	No statement	No	Cannot determine	Yes
Liberatoscioli *et al.,* 2011	Yes	Yes	Yes	Yes	Yes	No	Cannot determine	Yes
Maclennan *et al.,* 2012	No	Yes	No	Yes	No	No	Cannot determine	Yes
MacLeod *et al.,* 2012	No	Yes	No	No	No statement	No	Cannot determine	Yes
Miscoria *et al.,* 2010	Yes	Yes	Yes	Yes	No	No	Cannot determine	Yes
Mullard *et al.,* 2012	No	Yes	Yes	No	No statement	No	Cannot determine	Yes
Sim & Hayward, 2011	Yes	Yes	Yes	No	No statement	No	Cannot determine	Yes
Sparrow, 2011	No	Yes	No	No	No statement	No	Cannot determine	No
Susnerwala, 2011	No	Yes	Yes	No	No statement	Cannot determine	Cannot determine	Yes
Visvardis *et al.,* 201	No	Yes	Yes	No	No statement	No	Cannot determine	No
Wagstaff *et al.,* 2011	Yes	Yes	No	Yes	No statement	No	Cannot determine	No

Supplementary Table 2 Assessment of methodological quality of real world studies using the Chambers tool (Chambers *et al*., 2009).
All studies received a rating of poor according to the Chambers tool.
